# Improving coordination through information continuity: a framework for translational research

**DOI:** 10.1186/s12913-014-0590-5

**Published:** 2014-11-25

**Authors:** Karen Gardner, Michelle Banfield, Ian McRae, James Gillespie, Laurann Yen

**Affiliations:** Australian Primary Health Care Research Institute, The Australian National University, Cnr Mills & Eggleston Rds, Canberra, ACT 0200 Australia; Menzies Centre for Health Policy, School of Public Health, University of Sydney, Sydney, NSW 2006 Australia

**Keywords:** Coordination, Continuity, Information continuity, Primary health care, Translational research, Quality improvement

## Abstract

**Background:**

There is good evidence that coordination can have beneficial impacts on patient care and outcomes but the mechanisms by which coordination is to be achieved are poorly understood and rarely identified in relevant policies. One approach suggests that continuity of information is a key element but research is yet to provide guidance on how to optimise coordination through improving continuity in healthcare settings.

**Discussion:**

In this paper we report on the development of a conceptual framework of information continuity in care coordination. We drew on evidence from systematic reviews of coordination and empirical studies on information use in integrated care models to develop the framework. It identifies the architecture, processes and scope of practices that evidence suggests is required to support information continuity in a population based approach to care coordination.

**Summary:**

The framework offers value to policy makers and practitioners as a map that identifies the multi-level elements of an integrated system capable of driving better coordination. Testing of the framework in different settings could aid our understanding of information continuity as a mechanism for linking coordination strategies that operate at different levels of the health system and enable synthesis of findings for informing policy and practice.

## Background

Care coordination is a key feature of patient-centered health systems. There is good evidence that coordination can have beneficial impacts on care processes and patient outcomes [1-3] but reviews also reveal the term is inconsistently defined and used to refer to a range of strategies from those targeting the clinical care of individual patients to organisational and system interventions such as pooled funds, managed care and pay for performance schemes that provide incentives for achieving coordination [4-6]. These different interventions operate at micro (service), meso (primary health care organisations that facilitate links between health service providers and between organisations) and macro (system and policy) levels of the health system, but the mechanisms by which they achieve their outcomes are poorly understood [7] and lack of alignment between different levels impedes effective delivery [8]. Evidence from health systems around the world shows that organizational integration alone does not itself lead to improvements in coordination at the service level [2,9,10]. While meso level strategies like fund pooling and purchasing may be used to drive coordination for particular population groups for example, these must be aligned with micro level strategies like care planning, multidisciplinary teamwork, use of information technologies and disease management guidelines to improve outcomes [8].

From the patient perspective, continuity of care is the experience of care coordination [11]. Continuity refers to the degree to which care is linked and made coherent over time. Like coordination, continuity is associated with improvements in the quality of care and better outcomes such as higher satisfaction and lower hospitalization rates [12,13]. A recent review identified three separate dimensions: informational, management and relationship continuity, each of which is required to link discrete elements in a care pathway to achieve continuity [14].

Information is the common thread linking care from one provider to another and from one healthcare event to another [14]. Information continuity refers to the capacity of that information to travel with the patient and throughout the health system, between providers and over time, to facilitate a continuous care experience. This requires the organised collection of a patient’s information and relies on adequate medical records indicating episodes of illness, management and follow-up, as well as effective telecommunications, good referral systems, and feedback from other providers [12].

Continuity of care is usually taken to mean the relationship between a single practitioner and patient which transcends specific episodes of care. As primary health care moves further towards multidisciplinary teamwork however, efforts to achieve continuity will increasingly need to span multiple providers as well as multiple encounters over time. These and other changes in practice suggest that information continuity could be expected to play a central role in coordination since information can be made readily available and is conducive to automation [12]. Sustaining relationships between providers and their patients, ensuring that care is planned to meet needs and information flows when and where it is needed to support continuity is likely to present significant challenges [15].

This paper explores the intersection between information continuity and strategies for care coordination. It is part of a larger study examining coordination in Australian primary health care policy and practice [8,15,16]. In this paper, we draw on a review of systematic reviews of care coordination strategies conducted in the US for the Agency for Healthcare Research and Quality [1] and a review of clinical and service integration conducted for the Kings Fund in the UK [9] as well as our review of effective coordination strategies in Australia [8] to develop a conceptual framework of information continuity in care coordination that we propose may be used in translation research to strengthen policy and action responses. The framework postulates a multi-dimensional relationship in which information moves horizontally at the micro (service) level and vertically between the micro, meso and macro levels of the system to facilitate coordination. The framework has been developed with reference to the Australian system which suffers from extensive fragmentation arising in the division of responsibility for funding and delivering health between three levels of government. However, the framework may also have application in other countries where service integration and coordination of care are significant problems since the strategies it draws on are internationally recognized and their effectiveness have been demonstrated in multiple settings and countries.

## Discussion

### Interventions for achieving coordination

Interventions promoting coordination for long term conditions in primary health care include care planning, patient self management, case management, quality improvement processes at the micro level; development of regional organizational bodies with responsibility for service planning and devolved purchasing arrangements at the meso level; funding models and financial incentives at the macro level [8].

Models of coordination have operated largely at the patient level in Australia through strategies such as care planning and case conferencing which support the development of structured relationships between providers and patients and embed financial incentives for selected conditions within them [2]. A recent systematic review of evidence on the effectiveness of these strategies found that each was associated with improved health or patient satisfaction in more than 50% of the studies identified in the review [2]. Interventions that used multiple strategies were more effective than those relying on a single strategy.

Internationally, positive effects on patient outcomes from multidisciplinary teams, disease management, and case management across a number of clinical areas and topics managed in primary health care have also been observed [1]. In mental health, systematic reviews have shown that disease management programs can improve depression severity and adherence to treatment, and in patients with diabetes, disease management has reduced glycaeted haemoglobin and improved glycaemic control [1]. There is also some evidence that case management reduces re-hospitalization rates in patients with mental health problems and improves glycaemic control in patients with diabetes.

At the meso level, limited evidence is available from systematic reviews on the impact of organizational interventions for fundholding, purchasing or contracting that can be applied in the Australian context. Although organizational models can achieve change in the organization and delivery of primary health care they are reliant on the levers available to them to induce change, which primarily are funding and commissioning [17]. Four key domains for potential policy reform to increase coordination in Australia identified by Nacarella et al. were flexible GP funding; quality frameworks at a practice level; meso-level primary care organisations and investment in practice infrastructure [18].

Quality improvement strategies are part of the broader set of meso level programs, structures and incentives that provide the enabling factors capable of inducing improvement in the delivery of care at the micro level [2]. When used as part of structured quality improvement processes, patient education, provider education, provider reminders, audit and feedback, relay of clinical data, organizational change, financial and regulatory incentives, can be effective in changing professional practice [19,20], improving the quality of care [21-24] and patient outcomes [25].

At the macro level, pay-for-performance incentives have been used to stimulate improvements in a range of different services including coordination of care. A recent paper [25] based on a narrative review of evidence from five comparator countries [18] described pay-for-performance schemes as multi-faceted entities involving complex interventions that may include accreditation, education, quality improvement, investment in information technology and data collection systems, professional support and regional structures. Despite their popularity, the evidence relating to the impact of incentive payments on quality and outcomes is weak and unintended consequences have been documented in many models. Australia’s pay-for-performance approaches have been shaped by the absence of patient enrolment and rely on the use of practice based disease registers and fee-for-service activity captured in medicare payment systems through electronic patient record systems. Further development of automated data extraction at the practice level and incentive payments that reward team care and are based on completed annual cycles of care is needed [26].

### Information use in integrated models

Insights into how best to use information in these strategies to operationalize a coherent and multi-level approach to care coordination might best be drawn from studies of integrated care models in managed care organisations such as Kaiser Permanente which have demonstrated high performance against a number of indicators [9]. These models have historically used coordinating mechanisms to control costs, improve disease outcomes, quality of care, and patient satisfaction [1]. Information collected from patients about their medical condition, individual preference for care and personal and social circumstances is collated and used at different levels of aggregation to develop care arrangements, monitor quality and assess outcomes at different levels. Ham’s analysis of Kaiser Permanente’s performance point to multi-specialty group practice, aligned incentives, information technology and guidelines, accountability for performance and defined populations, a physician-management partnership, effective leadership and a collaborative culture as key success factors [9].

At the clinical level, integrated assessment and care planning use patient information to determine the scope, type and frequency of services to be provided. In practical terms this means using information to engage individuals and families in the redesign of their care; address complex multisystem issues for accessing services; provide planned care; facilitate referral, and co-manage care with multiple specialists based on evidence-based guidelines [27]. At the meso level, patient information is aggregated for analysis to assess and monitor improvements in disease outcomes, quality of care, patient satisfaction and program efficiencies.

### Information continuity in coordinated care

**Figure 1:**
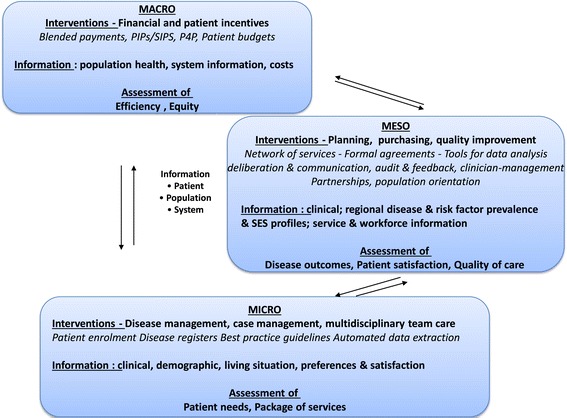
**Conceptual framework of information use in coordination of care.**

Bringing these different bodies of evidence together, we propose that information continuity operates in two dimensions to achieve coordination: horizontally at the clinical level to determine the scope, type and frequency of services required to link care events and ensure that these are connected and coherent; and vertically between mechanisms designed to stimulate coordination such as between financial incentives; quality improvement programs; care planning and case management interventions. Figure 1 reflects our synthesis of the evidence related to information use in care coordination. The framework aims to capture the breadth of information and use of evidence based tools, processes or strategies at different levels that can be used to improve continuity of care at the patient level and drive coordination across the system.

As shown in the diagram, information generated at the patient level about individual clinical and demographic characteristics, preferences and satisfaction may be used at that level to assess individual patient needs and packages of care. Use of disease registers, best practice guidelines, electronic records and automated data extraction tools support information continuity within case management and multi-disciplinary team care arrangements which promote coordination of care at that level.

Patient information may also be aggregated by population subgroup to assess disease outcomes or patient satisfaction at the meso level. When combined with risk factor prevalence, SES profile, workforce and other service information this data may also be used for quality improvement or to inform service planning and purchasing. Formal agreements between providers, tools for data analysis, deliberation and communication allow information to be analysed in different ways and repackaged for feed back to the patient level to support providers and patients in service improvement and patient self management initiatives. Thus group data from multiple services can provide the context in which individual services or patients are able to review their performance against self identified objectives.

At the macro level, the use of patient data together with cost and system information can be used to promote coordination for population subgroups through financial and practice incentives. However this is not routine in Australia and the development of patient budgets remains experimental. The difficulties associated with developing financial incentives to promote coordination of care for patient groups within the context of fee-for-service medicine and in the absence of patient enrolment have recently been demonstrated in the development of the Australian government Diabetes Pilot [26] and early results suggest that such developments will be iterative.

Notwithstanding these limitations, different types of information can be used to support continuity at the patient level and used to drive coordination across the system. Information can be aggregated and analysed in different ways and fed back in loops through different levels of organisation to assess questions related to patient needs and packages of care at the clinical level; disease outcomes, patient satisfaction and quality of care at the meso level; and equity and efficiency at the macro level.

## Summary

The framework offers value to policy makers and practitioners as a map that identifies the domains in which elements of an integrated system capable of driving better coordination need to be developed. Testing of the framework in different primary health care settings could aid our understanding of information continuity as a mechanism for linking coordination strategies that operate at different levels of the health system including how specific elements need to be enacted, implemented and sustained at the coalface. Such research will enable synthesis of findings for informing policy and practice.
